# Iatrogenic transmesenteric defect mimicking a Petersen’s space hernia after open pancreatic necrosectomy

**DOI:** 10.1093/jscr/rjae729

**Published:** 2024-11-24

**Authors:** Norman A Rendón Mejía, Alejandra Aguirre Aguilar, Carlos A Benítez Membrila, Pedro A Marquez Enriquez, David O Chora Rojas

**Affiliations:** Department of Surgery, Universidad Autonoma de Chihuahua, Hospital General de Chihuahua “Dr. Salvador Zubirán Anchondo”, Chihuahua 31200, México; Department of Surgery, Universidad Autonoma de Chihuahua, Hospital General de Chihuahua “Dr. Salvador Zubirán Anchondo”, Chihuahua 31200, México; Department of Surgery, Universidad Autonoma de Chihuahua, Hospital General de Chihuahua “Dr. Salvador Zubirán Anchondo”, Chihuahua 31200, México; Department of Surgery, Universidad Autonoma de Chihuahua, Hospital General de Chihuahua “Dr. Salvador Zubirán Anchondo”, Chihuahua 31200, México; Department of Surgery, Universidad Autonoma de Chihuahua, Hospital General de Chihuahua “Dr. Salvador Zubirán Anchondo”, Chihuahua 31200, México

**Keywords:** acute pancreatitis, necrosectomy, internal hernia, fistula

## Abstract

Complications of acute pancreatitis can be disastrous if appropriate treatment is not initiated. Pancreatic necrosis can occur without the presence of symptoms; however, in some cases, it can be accompanied by organic failure, abscess, pseudocyst, fistulas, and pancreatic exocrine disfunction. The surgical treatment of pancreatic necrosis can be managed with open surgical debridement of necrotic tissue. Hence, complications after surgery can appear even in patients without clinical background; complications mostly appear if the surgical technique is not done properly. We present a case of a 47-year-old woman who appeared with abdominal pain, nausea, vomiting, and oral intake intolerance. Symptoms were present for 1 week; she was admitted to the nearest clinic, and surgical management was offered. The patient went to an open pancreatic necrosectomy; however, she presented purulent exudate from the surgical wound and drains. Was referred to our center; on abdominal contrasted computed tomography, a transmesenteric defect and cutaneous-pancreatic fistula were found.

## Introduction

Acute pancreatitis is an inflammatory disease of exocrine pancreas that can lead to multiple organ dysfunction with a mortality of 1%–5%. About two-thirds of patients with acute pancreatitis will have an uncomplicated course, with resolution of symptoms within days [1]. The presence of necrosis in the onset of acute pancreatitis may develop in 20% of cases; necrosis is diagnosed if pancreatic tissue does not enhance after contrast administration on a computer tomography scan [2]. Pancreatic necrosis is an indication for operative debridement; pancreatic necrosectomy has been considered in patients who are symptomatic and used where no other options are available. Open necrosectomy consists of an upper midline incision, lesser sac, and mesocolon mobilization in an avascular plane between the omentum and transverse colon. Commonly transverse mesocolon is preserved to remain as a barrier and protect the inferior abdominal contents from contamination [3].

Early complications from open necrosectomy include: organ failure, portal and splenic vein thrombosis, pneumonia, colonic necrosis, gastrointestinal fistulae, hemorrhage, and fungal infection. However, late complications from open necrosectomy are biliary stricture, pseudocyst, pancreatic fistula, gastrointestinal fistula, collections, incisional hernia, exocrine insufficiency, and diabetes mellitus [4]. Postoperative pancreatic fistula represents a parenchymal leak from the pancreatic ductal system into around the pancreas and not necessarily to another epithelized surface [5]. Commonly, pseudocysts develop from pancreatic large duct occlusion causing smaller duct obstruction and dilatation; rarely, pseudocysts develop after trauma-related ductal disruption or after surgical injury [6]. Postoperative incisional hernias after open necrosectomy usually develop from dehiscence of abdominal wall muscles due to pancreatic leak and fistulous tract formation. Hence, the development of internal hernias after open pancreatic necrosectomy has not been reported in medical literature. Postoperative internal hernias have been present on bariatric surgery and liver transplantation; protrusion of abdominal viscera, most commonly small bowel loops, through a peritoneal or mesenteric aperture into the retroperitoneum has been reported [7]. This defect on mesentery is found near the Treitz ligament in association with the transverse mesocolon; this space is historically known as Petersen’s space; the herniary content from this ring consists of a 90° small bowel loop torsion and an efferent limb from Roux-en-Y anastomosis [8].

We present a 47-year-old woman who developed a transmesenteric defect complicated by a cutaneous-pancreatic fistula after an open pancreatic necrosectomy.

## Case presentation

We present a case of a 47-year-old woman who presented abdominal pain, distension, nausea, vomiting, and oral intake intolerance for 1 week. She attends the nearest medical center; during clinical evaluation, mesogastrium and right hypochondrium pain were found after deep palpation. A simple abdominal computed tomography (CT) was made; pancreatic parenchyma with evident increased size with two peripancreatic fluid collections on the body and head of the pancreas were found. Acute care surgeon decides surgical debridement; an upper midline abdominal incision was made; free fluid was observed on the greater omentum and left parietocolic space; omental transcavity was reached through the transverse mesocolon window; necrotic collections were found on the body and head of the pancreas, devitalized tissue was evacuated; and a surgical drain was placed within the body of pancreas. Aggressive fluid therapy, intravenous broad-spectrum antibiotics, total parenteral nutrition, and supplementary oxygen were administered.

**Figure 1:**
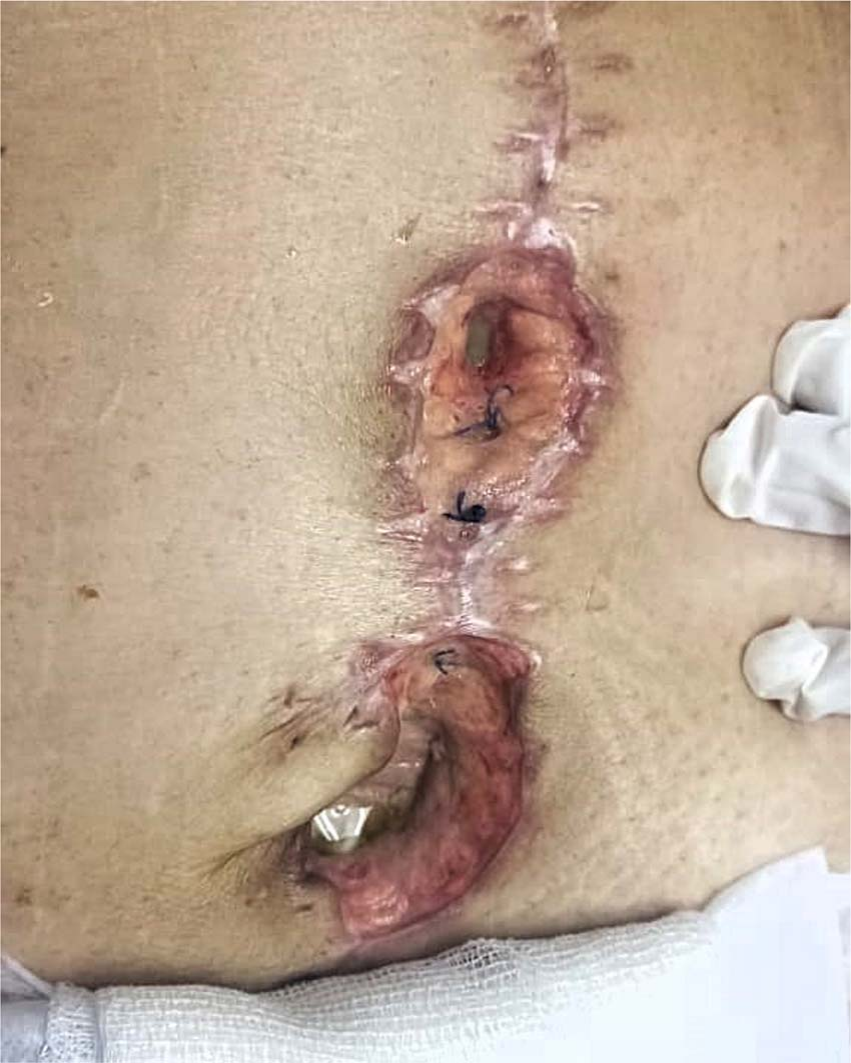
Dehiscence of abdominal surgical wound, inside view of infected aponeurotic layer with purulent exudate from the inferior border of the wound.

**Figure 2:**
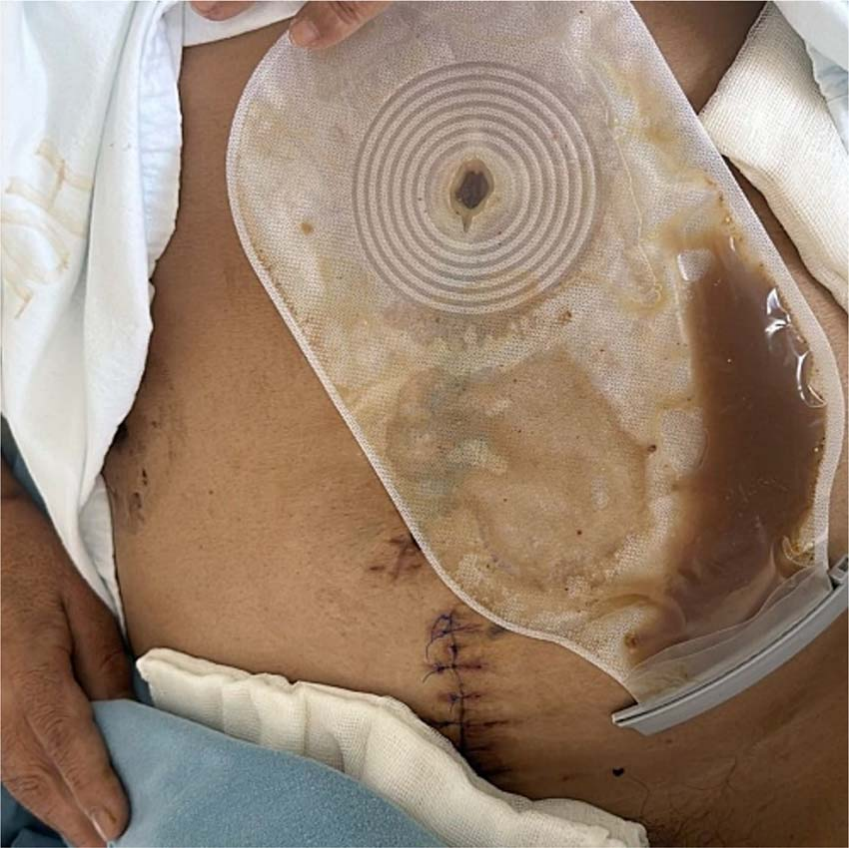
Cutaneous pancreatic fistula with abundant purulent-brownish exudate from midline laparotomy wound.

On postoperative day (POD) 3, the patient developed refractory hypertension, and surgical wound dehiscence was found in the epigastrium with abundant purulent exudate (Fig. 1). Surgical wound cultures were taken, and bedside debridement was done due to continuous abundant purulent exudate. Amylase levels were measured from surgical wound exudate, resulting with three times-fold higher values than normal values (Fig. 2). Surgical exploration and lavage were intended. A midline incision was made from the previous incision, 1 L of free serous-hematic fluid was found; small and large bowel loops with loss of normal anatomy; a fistulous tract was identified on the retroperitoneum; no debridement was possible.

**Figure 3:**
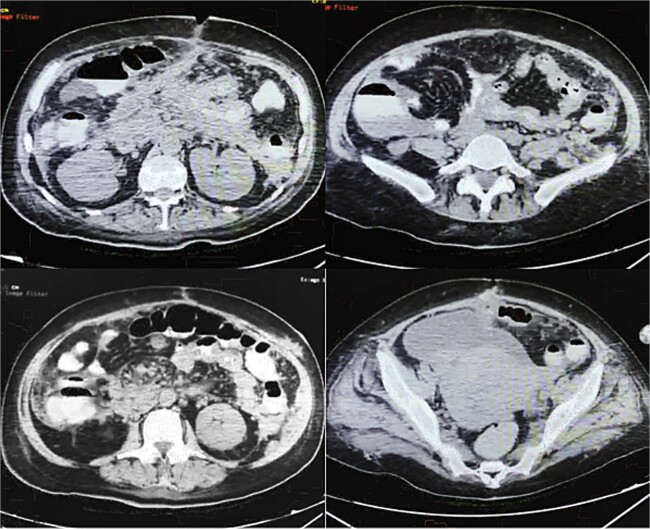
Oral contrasted abdominal CT with presence of abundant pancreatic collections on body and tail of pancreas with a volume of 132 ml, with evident fistulous tract from pancreatic parenchyma to abdominal fat and muscles near the surgical wound, torsion of mesenteric fat and vessels and terminal ileum loops in right abdominal space, free abdominal fluid on pelvic space.

On POD 14, an oral contrasted abdominal CT scan was solicited; multiple pancreatic collections, the presence of a pseudocyst on the body of pancreas, an abscess on the tail of the pancreas, a fistulous tract from the pancreatic parenchyma directed to the surgical wound, and mesenteric vessels with counter clock-wise torsion below the transverse mesocolon (whirl sign) with small bowel loops near the ileocecal valve were observed (Fig. 3).

## Discussion

Most patients with acute pancreatitis have an uncomplicated course, with symptoms resolution within days; however, in 10% of cases, patients develop severe acute pancreatitis, accompanied with more prolonged severe abdominal pain, nutritional deficit, and hospital stays [1]. In our case, the patient developed pancreatic necrosis and prolonged stay due to oral intake intolerance. Hence, infection of necrotic collections increases morbidity and mortality rates, which requires a drainage procedure [2].

However, selecting an appropriate surgical approach can directly benefit patient clinical outcomes. Open surgical necrosectomy is considered a safe approach with outstanding results; moreover, a minimally invasive approach has been considered the modality of choice due to better outcomes [3, 4]. Complications from open necrosectomy usually develop after 4–6 weeks with biliary stricture, pseudocyst, pancreatic fistula, gastrointestinal fistula, delayed collections, incisional hernia, exocrine insufficiency, and diabetes mellitus [4].

Rare complications from open necrosectomy may be explained by a decreased number of open procedures and increased minimal invasive procedures; better outcomes and less mortality have been reported in recent years. However, in low resource settings, endoscopic and laparoscopic drainage are not always available; increased costs and lack of equipment for these procedures are the most common causes [3–7]. Our patient after open necrosectomy had a transient improvement of symptoms; however, complications appeared in an early phase (<4–6 weeks). On CT images, structural complications from the first procedure were found, but the presence of a defect on the mesentery compatible with an internal hernia was unexpected.

Hernial orifices can be acquired, caused by inflammation, trauma, or previous surgery, like gastric bypass in bariatric surgery and liver transplantation; transmesenteric hernias account for ~8% of all internal hernias [7]. Usually, these defects appear after bariatric procedures; the explanation for this event is related to the creation of a surgical window through mesentery to descend Roux-en-Y efferent limb [8].

Currently in medical literature, there is no other case of simultaneous complications, including transmesenteric defect associated with a cutaneous-pancreatic fistula. These findings may be explained by a low-experienced surgeon and the absence of resources for a minimal invasive approach. Petersen’s hernia properly develops from a mesentery defect after gastrojejunostomy; moreover, in our case we can’t classify this finding into this category, despite the similarities from the CT images and clinical findings [6–8].

## References

[1] Szatmary P, Grammatikopoulos T, Cai W, et al. Acute pancreatitis: diagnosis and treatment. Drugs 2022;82:1251–76. 10.1007/s40265-022-01766-4. 36074322 PMC9454414

[2] Leonard-Murali S, Lezotte J, Kalu R, et al. Necrotizing pancreatitis: a review for the acute care surgeon. Am J Surg 2021;221:927–34. 10.1016/j.amjsurg.2020.08.027. 32878690 PMC8650167

[3] Traverso LW, Kozarek RA. Pancreatic necrosectomy: definitions and technique. J Gastrointest Surg 2005;9:436–9. 10.1016/j.gassur.2004.05.013. 15749608

[4] Connor S, Alexakis N, Raraty MGT, et al. Early and late complications after pancreatic necrosectomy. Surgery 2005;137:499–505. 10.1016/j.surg.2005.01.003. 15855920

[5] Bassi C, Dervenis C, Butturini G, et al. Postoperative pancreatic fistula: an international study group (ISGPF) definition. Surgery 2005;138:8–13. 10.1016/j.surg.2005.05.001. 16003309

[6] Byrne MF, Mitchell RM, Baillie J. Pancreatic pseudocysts. Curr Treat Options Gastroenterol 2002;5:331–8. 10.1007/s11938-002-0021-2. 12207856

[7] Lanzetta MM, Masserelli A, Addeo G, et al. Internal hernias: a difficult diagnostic challenge. Review of CT signs and clinical findings. Acta Biomed 2019;90:20–37. 10.23750/abm.v90i5-S.8344. PMC6625567 31085971

[8] Rogers AM, Ionescu AM, Pauli EM, et al. When is a Petersen’s hernia not a Petersen’s hernia. J Am Coll Surg 2008;207:121–4. 10.1016/j.jamcollsurg.2008.01.019. 18589370

